# Characterization of the Buccula, Rostrum, Stridulatory Sulcus, Scutellum, and External Female Genitalia of* Triatoma carcavalloi* (Jurberg, Rocha & Lent, 1998),* Triatoma circummaculata* (Stål, 1859), and* Triatoma rubrovaria* (Blanchard, 1843) (Hemiptera, Reduviidae, Triatominae)

**DOI:** 10.1155/2019/3517098

**Published:** 2019-07-22

**Authors:** Margareth Alves Ribeiro Cardozo de Almeida, Simone Patrícia Carneiro Freitas, Maria Luiza Ribeiro de Oliveira, Nathanielly Rocha Casado de Lima, Elizabeth Ferreira Rangel, Jacenir Reis Santos-Mallet

**Affiliations:** ^1^Laboratório Interdisciplinar de Vigilância Entomológica em Diptera e Hemiptera, Instituto Oswaldo Cruz, FIOCRUZ, Av. Brasil 4365, Pavilhão Carlos Chagas, 5° Andar, Rio de Janeiro, RJ 21040-360, Brazil; ^2^Bolsista Treinamento e Capacitação Técnica, FAPERJ, Av Erasmo Braga 118, Rio de Janeiro, RJ 20020-000, Brazil; ^3^Laboratório de Biodiversidade Entomológica, Instituto Oswaldo Cruz, FIOCRUZ, Av. Brasil 4365. Pavilhão Mourisco, Sala 214, Rio de Janeiro, RJ 21040-360, Brazil

## Abstract

In Brazil,* Triatoma rubrovaria *(Blanchard, 1843) is the most important species in epidemiological terms in the State of Rio Grande do Sul, due to its wide geographical distribution in this state, followed by* T. carcavalloi* (Jurberg, Rocha & Lent, 1998) and* T. circummaculata *(Stål, 1859). Structural analysis of the ventral region of the head (rostrum and buccula), thorax (stridulatorium sulcus and scutellum), and external female genitalia of adults of* T. rubrovaria*,* T. carcavalloi*, and* T. circummaculata* is described here. Scutellum, head, rostrum, and part of the thorax (prosternum) containing the stridulatory sulcus, in both male and female, and the sixth abdominal segment of the female, containing the external genitalia, were processed for scanning electron microscopy studies as routine. Morphological differences in the analyzed structures for all the three* Triatoma *species studied were detected under scanning electron microscopy. This study confirms the grouping of the* T. rubrovaria, T. carcavalloi, *and* T. circummaculata* in ‘*T. rubrovaria *subcomplex' by their morphological similarities.

## 1. Introduction

Chagas disease is a parasitic, systemic, and chronic disease caused by the protozoan* Trypanosoma cruzi* (Chagas, 1909) (Kinetoplastida, Trypanosomatidae), with risk factors strongly associated with low socioeconomic factors, besides being considered a neglected and endemic tropical disease in 21 countries of the Americas. This disease is mainly transmitted through the feces of triatomines. These insects have, as main biological characteristic, hematophagy in all phases of nymph and adult [1].

Currently, the subfamily Triatominae is composed of 154 species [2–7], which are all potential transmitters of Chagas disease. In Brazil,* T. rubrovaria* (Blanchard, 1843) is the most important species in epidemiological terms in the State of Rio Grande do Sul, due to its wide geographical distribution in this state, followed by* Triatoma carcavalloi* (Jurberg, Rocha & Lent, 1998) and* Triatoma circummaculata *(Stål, 1859). These species have wild habits, live in sympatry, and invade the human home frequently, due to changes in environments produced by anthropic activities, as well as the elimination of* Triatoma infestans* (Klug, 1834) from the domicile leaving available niche [8, 9].

The taxonomy of Triatominae is based on the external morphological characters and indicated the importance of the stridulatory sulcus, which varies in the form, length, number, and space of the sulcus [10, 11]. The importance of the rostrum in this subfamily has been underlined since the 1920s [12].

In cytogenetic, molecular, and morphometric analyses of the head, including the antennas, thorax, and abdomen, it was demonstrated that it is possible to distinguish completely between* T. maculata *(Erichson, 1848)*, T. pseudomaculata* (Corrêa & Espínola, 1964), and* T. arthurneivai* (Lent & Martins, 1940), with the evolutionary relation of the first species, in relation to the last ones, being questioned [13–15]. Isoenzymatic and chromatic results corroborate each other and favor the hypothesis of a distinct and isolated population* T. rubrovaria *[16]. Study of the female genitalia by scanning microscopy showed that the description of such characters may be valuable for a definition of more specific species, contributing to the phylogenetic and taxonomic study in subfamily Triatominae [17].

*Triatoma *(Laporte, 1832) genus presents specific patterns in scutellum, not only in shape and length, but also in the cuticular structure, central depressions, projections, and processes that allow its use in species differentiation [18]. The use of scanning electron microscopy (SEM) has helped to clarify many studies on external morphology in triatomines: details on the morphology of abdominal bristles [19]; shape and proportions of scutellum in* T. ryckmani *Zeledón & Ponce, 1972 [20]; characterization the scutellum of* T. guazu *Lent & Wygodzinsky, 1979 [21] and other eight species of the* Triatoma *[22] and six* Meccus *Stål, 1859, species [23]; and important contributions for the study of nymphs [24].

In the last years, the external genitalia of females have been studied in more detail with the use of SEM. The use of this tool made it possible to characterize several species of the subfamily Triatominae [17, 25–29]. This is the first morphological description of the buccula, rostrum, stridulatory sulcus, scutellum, and external female genitalia in adults of* T. rubrovaria*,* T. carcavalloi*, and* T. circummaculata* using SEM, showing clear distinctions in relation to those characters.

## 2. Materials and Methods

The thirty specimens (five males and five females of each species) were obtained from colonies started with specimens collected in the field and domiciliary of the municipality of Encruzilhada do Sul, State of Rio Grande do Sul (RS) (30°32′38′′S; 52°31′19′′O), and maintained at 26°C and 70% RH (relative humidity) at the Laboratório Interdisciplinar de Vigilância Entomológica em Diptera e Hemiptera, Instituto Oswaldo Cruz, FIOCRUZ, Rio de Janeiro.

For the ultrastructural analysis, we separated the scutellum, the head, the rostrum, and part of the thorax (prosternum) containing the stridulatory sulcus, in both male and female, and the sixth abdominal segment of the female, containing the external genitalia. These structures were washed in distilled water and dehydrated in increasing alcoholic series at the concentrations of 7.5%, 15%, 30%, 50%, 70%, 90%, and (3 times for) 100% by immersion for 10 minutes at each concentration.

The structures were mounted in aluminium stubs adhered to an adhesive double-sided tape and left in the incubator at 60°C for 2 hours for drying and in a desiccator containing silica gel until metallization. The structures after drying were pulverized with gold and later and the analysis was performed by the Scanning Electron Microscope JEOL 6390LV of the Electron Microscopy Platform, Instituto Oswaldo Cruz, FIOCRUZ.

## 3. Results and Discussion

Until the 1960s, the taxonomy of triatomines was used as basic criterion for external and chromatic morphological characters, but during the last decade, SEM has been used as an important tool for Triatominae systematics, justifying the status of cryptic species and their complexes [11, 25, 27, 28, 30, 31].

### 3.1. Buccula, Rostrum, and Stridulatory Sulcus

**Figure 1:**
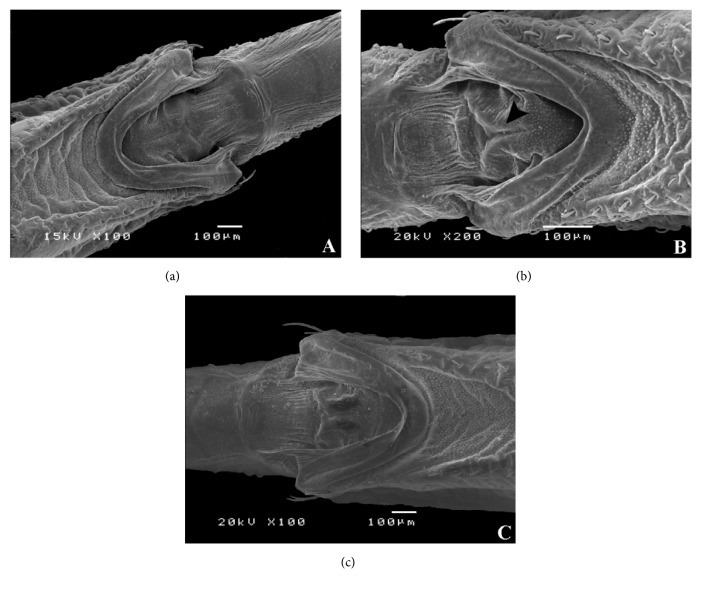
Micrography of the buccula. (a)* Triatoma carcavalloi *male (U-shaped). (b)* Triatoma circummaculata* male (V-shaped). (c)* Triatoma rubrovaria* male (U-shaped). Rift (arrow head).

The buccula of the* T. carcavalloi *(Figure 1(a)),* T. circummaculata* (Figure 1(b)), and* T. rubrovaria *(Figure 1(c)) is located in the posterior region of the rostrum, with a thick edge on the anterior region and granular surface. In the three species, the anterior region shows accentuated pleats in both females and males. The buccula of the* T. carcavalloi* (Figure 1(a)) and* T. rubrovaria *(Figure 1(c)) is U-shaped, while that of* T. circummaculata* is V-shaped (Figure 1(b)); it can thus be considered a character used as a diagnosis to differentiate this species from the others of the ‘*T. rubrovaria* subcomplex' [11]. The buccula form seems to be the same among the triatomines already studied, since* T*.* klugi *and* T*.* vandae* [32],* T*.* guazu* and* T*.* jurbergi* [30] presented the U-shaped, according to the* T*.* carcavalloi* and* T*.* rubrovaria* observed in our studies.

In* T. carcavalloi* and* T. rubrovaria*, the internal area of the central region presented a rift which can be considered as characteristic of sexual dimorphism, since it is only present in the females and absent in males (Figures 1(a) and 1(c)) [11]. In* T. circummaculata*, a rift is present in females and males (Figure 1(b)).

**Figure 2:**
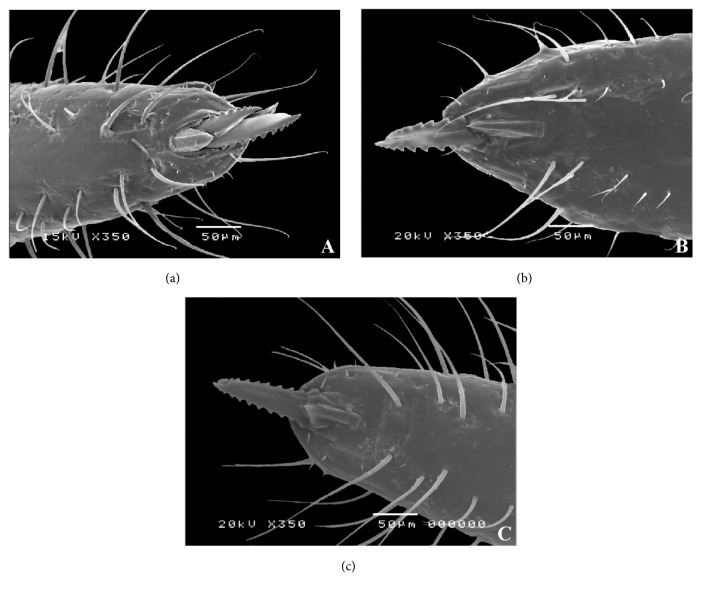
Micrography of the apex rostrum male. (a)* Triatoma carcavalloi*. (b)* Triatoma circummaculata*. (c)* Triatoma rubrovaria*.

The rostrum is similar in both sexes in the three species. The apical plate has a lozenge formed inferior lamella and the superior one has a digit form. Two lateral rifts 1+1 were observed at the apex of the rostrum (Figure 2).

**Figure 3:**
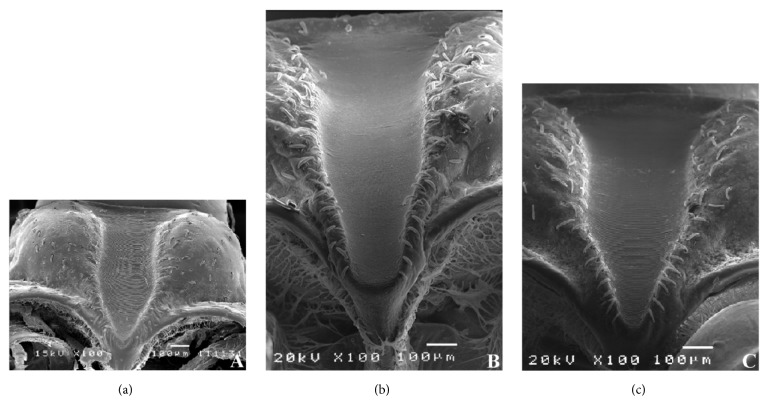
Micrography of the stridulatory sulcus. (a)* Triatoma carcavalloi *male (V-shaped). (b)* Triatoma circummaculata *female (U-shaped). (c)* Triatoma rubrovaria *male (V-shaped).

The stridulatory sulcus of the* T. carcavalloi* and* T. rubrovaria *is V-shaped (Figures 3(a) and 3(c), respectively) and that of* T. circummaculata* is U-shaped (Figure 3(b)). In the three species, the posterior edge and the acetabulum cavity present depression in the central region, with marked parallel striae delimited by sensillae. This structure is larger in females than males.

The stridulatory sulcus is an important structure in the identification of the specie [10, 11]. This structure was appropriate to separating the species of* T. maculata* and* T. pseudomaculata* [33]. In a study of the* T. williami* and* T. klugi*, nymphs of 3rd instar differ on ornamentation and quantity of rifts in the internal central area of the stridulatory sulcus [32]. In our studies, the stridulatory sulcus can also be used to separate* T. carcavalloi, T. circummaculata*, and* T. rubrovaria* species.

### 3.2. Scutellum

**Figure 4:**
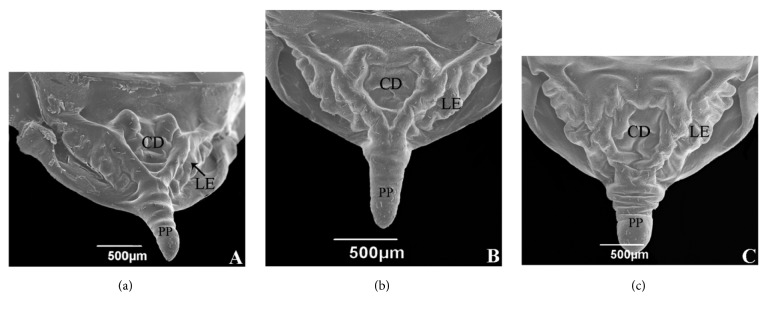
Micrography of the scutellum female. (a)* Triatoma carcavalloi.* (b)* Triatoma circummaculata*. (c)* Triatoma rubrovaria*. CD: central depression. LE: lateral edges. PP: posterior process of scutellum.

The scutellum of the three species had a triangular shape with sensilla distribution (Figure 4). The lateral edges are heavily sculpted and irregular (Figure 4). The shapes of central depression in* T. carcavalloi *and* T. rubrovaria* are similar, being W-shaped (Figures 4(a) and 4(c)). Already* T. circummaculata* is cordiform (heart-shaped) (Figure 4(b)). In* T. rubrovaria*, the posterior process scutellum is cylindrical and shorter than in* T. carcavalloi* and* T. circummaculata* and presents transverse striations (Figure 4(c)). Comparing our results with those obtained by Obara et al. [22], we find that the scutellum of* T*.* vandae* is very similar to that of* T*.* carcavalloi*, differing only in the arrangement of the sensillae of the posterior process.* T*.* carcavalloi* does not present sensilla in the central region.

A morphological analysis of the scutellum of eight species belonging to the genus* Triatoma* revealed important characteristics, especially the form of central depression and the posterior scutellar process [22]. Of all the species studied, four presented the form of cordiform central depression. This form appears to be a generic characteristic of this group, which agrees with literature data, as most species analyzed so far exhibit this pattern. However, some exceptions have been found, such as in* T*.* tibiamaculata*,* T*.* eratyrusiformis*, and* T*.* sherlocki* [22]. In our studies we performed a morphological study of the scutellum of* T*.* carcavalloi*,* T*.* circummaculata*, and* T*.* rubrovaria*. Of these three species only one presented the cordiform form,* T. circummaculata *(which can be used to differentiate it from the ‘*T. rubrovaria* subcomplex' species) [Table 1]. In addition, we can differentiate the forms of the scutellar regions and greater or lesser concentration of sensilla proving to be taxonomically important.

### 3.3. Female External Genitalia

**Figure 5:**
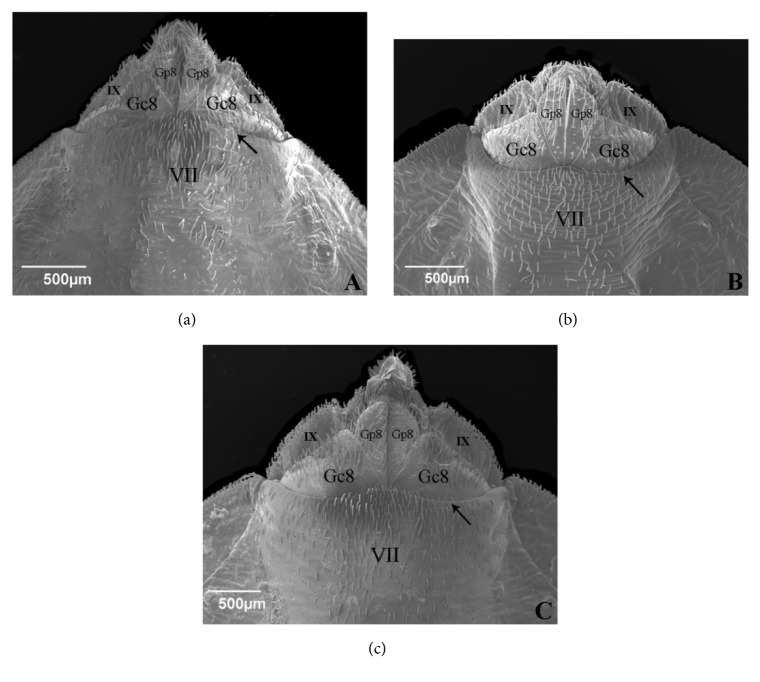
Ventral view of external female genitalia. (a)* Triatoma carcavalloi.* (b)* Triatoma circummaculata.* (c)* Triatoma rubrovaria.* Gc8: gonocoxite 8. Gp8: gonapophyses 8. VII: sternite 7: IX: sternite 9. Line separating the VII sternite and the pair of VIII gonocoxites (arrow).

In ventral side the line separating the VII sternite and the pair of VIII gonocoxites has greater curvature in* T. rubrovaria* than in* T. carcavalloi* and* T. circummaculata* (Figure 5). In the three species, the VIII gonocoxites are large and have a subtriangular shape, and are wider and long in* T. carcavalloi* and* T. rubrovaria* (Figures 5(a) and 5(c), respectively) than* T. circummaculata* (Figure 5(b)). The base of VIII gonapophyses of* T. carcavalloi* presents a triangular shape, which differs from* T. circummaculata* and* T. rubrovaria* which are more extended. There is a difference in the length of the VIII gonapophyses of these three species; in* T. circummaculata* it is much smaller. The lateral expansions of IX sternite are perceptible in all three species and follow the same format as the VIII gonocoxites (Figure 5).

The study of the external morphology of females in triatomines was reevaluated, when it verified the relevant taxonomic differences in the genitalia of the females through scanning electron microscopy, which allowed the differentiation of the species of the genera* Panstrongylus*,* Rhodnius*, and* Triatoma* as well as the taxonomic identification of 12 species of* Rhodnius*, whose identification by general external morphology causes doubts [17]. In this work, the use of the same methodology also allowed the separation between* T*.* carcavalloi*,* T*.* circummaculata*, and* T*.* rubrovaria* species that live in the same ecotype and are very morphologically similar.

**Figure 6:**
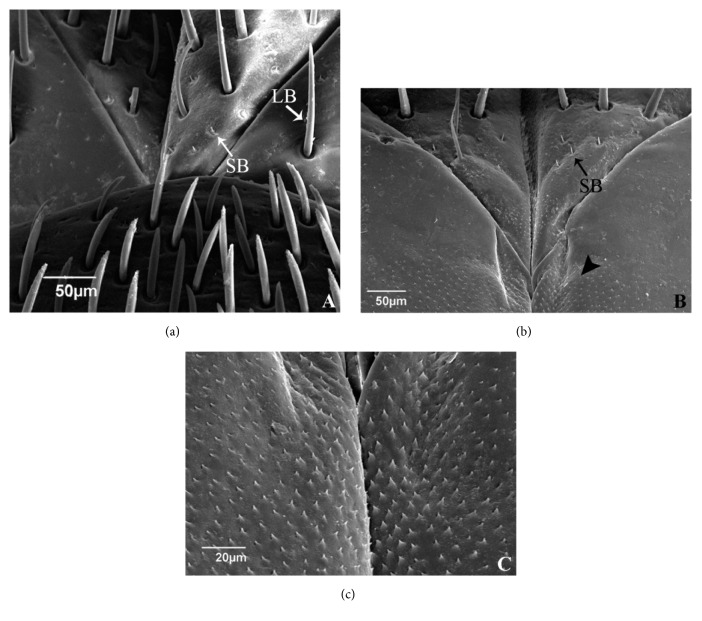
(a) VIII gonapophyses of the* Triatoma carcavalloi *with short bristles (SB) and long bristles (LB). (b) Short bristles (SB) and cuticular structures similar to spines (arrowhead) in VIII gonapophyses of the* Triatoma circummaculata*. (c) Cuticular structures similar to spines (arrowhead) in VIII gonapophyses of the* Triatoma rubrovaria*.

In the three species, the base of VIII gonapophyses shows short and smooth bristles, whereas in the other segments of the genitalia, long and fluted bristles are seen (Figures 6(a) and 6(b)), but only in* T. rubrovaria*, besides the bristles are seen cuticular structures similar to spines (Figures 6(b) and 6(c)).

These results reinforce the status of* T*.* carcavalloi*,* T*.* circummaculata*, and* T*.* rubrovaria* in the ‘*T. rubrovaria *subcomplex', conceptualize the morphological differences of these species, and contribute to an earlier diagnosis in the endemic areas of Chagas disease in Rio Grande do Sul.

## Figures and Tables

**Table 1:** Characterization of the buccula, rostrum, stridulatory sulcus, scutellum, and female genital of *Triatoma* species studied.

Species	Structures				
	Buccula	Rostrum	Stridulatory sulcus	Scutellum	Female genital
*T. carcavalloi*	U-shaped	Two lateral rifts 1+1	V-shaped	W-shaped	Smaller curvature (VII sternite and VIII gonocoxite)
*T. circummaculata*	V-shaped	Two lateral rifts 1+1	U-shaped	Cordiform	Smaller curvature (VII sternite and VIII gonocoxite)
*T. rubrovaria*	U-shaped	Two lateral rifts 1+1	V-shaped	W-shaped	Greater curvature (VII sternite and VIII gonocoxite)
*T. costalimai* [22]	-	-	-	Cordiform	-
*T. delpontei *[22]	-	-	-	Cordiform	-
*T. infestans *[22]	-	-	-	Cordiform	-
*T. guazu *[30]	U-shaped	-	-	-	-
*T. jurbergi* [30]	U-shaped	-	-	-	-
*T. klugi *[32]	U-shaped	Two lateral rifts 1+1	V-shaped	-	-
*T. vandae *[22, 32]	U-shaped	Two lateral rifts 1+1	V-shaped	Cordiform	-
*T. williami *[32]	U-shaped	Two lateral rifts 1+1	V-shaped	-	-
*T. maculata *[33]	-	-	V-shaped	-	-
*T. pseudomaculata *[33]	-	-	V-shaped	-	-

## Data Availability

The main part of the data generated or analyzed during this study is included in this published article. Other data will be available from the corresponding author upon request.
